# Dichloridobis(2-{1-[2-(1*H*-indol-3-yl)ethyl­iminio]eth­yl}phenolate-κ*O*)zinc(II)–2-{1-[2-(1*H*-indol-3-yl)ethyl­iminio]eth­yl}phenolate (1/2)

**DOI:** 10.1107/S1600536808011161

**Published:** 2008-04-26

**Authors:** Hapipah M. Ali, Mohamed Ibrahim Mohamed Mustafa, Mohd. Razali Rizal, Seik Weng Ng

**Affiliations:** a Department of Chemistry, University of Malaya, 50603 Kuala Lumpur, Malaysia

## Abstract

In the mononuclear complex mol­ecule of the title compond, [ZnCl_2_(C_18_H_18_N_2_O)_2_]·2C_18_H_18_N_2_O, the Zn atom, which lies on a twofold rotation axis, is coordinated by phenolate O atoms in a tetra­hedral coordination geometry. The coordinated Schiff base uses its indole NH donor site to form a hydrogen bond to the negatively charged phenolate O atom of the uncoordinated zwitterionic Schiff base. There is an intra­molecular N—H⋯O hydrogen bond in the coordinated and uncoordinated Schiff bases. The indole NH site of the uncoordinated Schiff base does not engage in a hydrogen-bond inter­action. The CH_2_—CH_2_ group in the uncoordinated Schiff base is disordered equally over two positions.

## Related literature

For a related neutral Schiff base, see: Rodriguez *et al.* (1987 ▶). For a related but zwitterionic Schiff base, see: Ali *et al.* (2007 ▶). For zinc derivatives of such deprotonated Schiff bases, see: Ali *et al.* (2008 ▶); Chen *et al.* (2007 ▶).
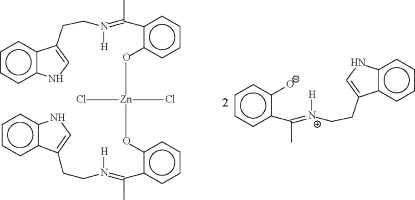


## Experimental

### 

#### Crystal data

[ZnCl_2_(C_18_H_18_N_2_O)_2_]·2C_18_H_18_N_2_O
*M* *_r_* = 1249.65
Monoclinic, 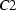

*a* = 25.8073 (4) Å
*b* = 9.1754 (1) Å
*c* = 14.3265 (2) Å
β = 112.566 (1)°
*V* = 3132.67 (7) Å^3^
*Z* = 2
Mo *K*α radiation
μ = 0.54 mm^−1^
*T* = 295 (2) K
0.10 × 0.06 × 0.05 mm

#### Data collection

Bruker SMART APEX diffractometer
Absorption correction: multi-scan (*SADABS*; Sheldrick, 1996 ▶) *T*_min_ = 0.866, *T*_max_ = 0.974
36333 measured reflections
7190 independent reflections
6008 reflections with *I* > 2σ(*I*)
*R*_int_ = 0.039

#### Refinement

*R*[*F* ^2^ > 2σ(*F* ^2^)] = 0.037
*wR*(*F* ^2^) = 0.091
*S* = 1.00
7190 reflections
413 parameters
10 restraints
H atoms treated by a mixture of independent and constrained refinement
Δρ_max_ = 0.35 e Å^−3^
Δρ_min_ = −0.30 e Å^−3^
Absolute structure: Flack (1983 ▶), 3669 Friedel pairs
Flack parameter: 0.000 (8)

### 

Data collection: *APEX2* (Bruker, 2007 ▶); cell refinement: *SAINT* (Bruker, 2007 ▶); data reduction: *SAINT*; program(s) used to solve structure: *SHELXS97* (Sheldrick, 2008 ▶); program(s) used to refine structure: *SHELXL97* (Sheldrick, 2008 ▶); molecular graphics: *X-SEED* (Barbour, 2001 ▶); software used to prepare material for publication: *publCIF* (Westrip, 2008 ▶).

## Supplementary Material

Crystal structure: contains datablocks global, I. DOI: 10.1107/S1600536808011161/sg2225sup1.cif

Structure factors: contains datablocks I. DOI: 10.1107/S1600536808011161/sg2225Isup2.hkl

Additional supplementary materials:  crystallographic information; 3D view; checkCIF report

## Figures and Tables

**Table:** 

Zn1—O1	1.987 (2)
Zn1—Cl1	2.2260 (6)

**Table:** 

O1—Zn1—O1^i^	99.84 (9)
O1—Zn1—Cl1	110.51 (5)
O1—Zn1—Cl1^i^	110.14 (5)
Cl1—Zn1—Cl1^i^	114.72 (3)

Symmetry code: (i) 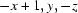.

**Table 2:** Hydrogen-bond geometry (Å, °)

*D*—H⋯*A*	*D*—H	H⋯*A*	*D*⋯*A*	*D*—H⋯*A*
N1—H1*n*⋯O1	0.86 (1)	1.81 (2)	2.557 (3)	144 (3)
N2—H2*n*⋯O2	0.86 (1)	2.01 (1)	2.851 (3)	164 (3)
N3—H3*n*⋯O2	0.87 (1)	1.83 (3)	2.503 (4)	133 (4)

Symmetry codes: .

**Fig. 1.**
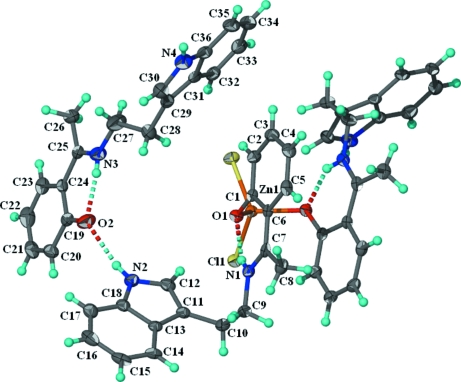
Thermal ellipsoid plot of ZnCl2(C18H18N2O)2.2C18H18N2O at the 70% probability level; hydrogen atoms are drawn as spheres of arbitrary radiius. The symmetry-related zwitterionic Schiff base is not shown. Dashed lines denote hydrogen bonds. The mononuclear molecule lies on a twofold rotation axis.

## supplementary crystallographic information

### Comment

The reaction of zinc acetate with 2-[2-(1*H*-indol-3-yl)ethyliminomethy]phenol, a neutral Schiff base (Rodriguez *et al.*, 1987) furnishes the expected zinc complex with the deprotonated ligand in which the deprotonated ligand N,*O*-chelates to the metal center (Chen *et al.*, 2007). Similarly, the reaction of zinc acetate with the 4-methyl substituted Schiff base (the methyl substituent is *para* to the negatively-charged phenoxy group) affords the corresponding tetrahdral zinc compound; the structure of the Schiff base itself is not known.

On the other hand, the 4-methyl substituted Schiff base of 2-[2-(1*H*-indol-3-yl)ethylimino-1-ethy]phenol exists in the zwitterionic form; the presence of methyl group on the imino –C=N– double-bond probably induces charge separation (Ali *et al.*, 2007). 2-[2-(1*H*-Indol-3-yl)ethylimino-1-ethy]phenol in the title cocrystal is a zwitterionic species; it coordinates to zinc chloride, so that the coordination geometry at the metal center is an Cl_2_O_2_Zn tetraheron. The compound crystallizes as a cocrystal (Scheme I, Fig. 1). Both the coordinated and free zwitterionis have an intramolecular N···O hydrogen bond. The coordinated Schiff base uses its N–H_indolyl_ donor site to form a hydrogen bond to the negatively-charged phenolato-O atom of the free Schiff base. The N–H_indolyl_ site of the free Schiff base does not engage in a hydrogen-bonding interaction.

### Experimental

The Schiff base was synthesized by condensing 2-(1*H*-indol-3-yl)ethylamine with 2-hydroxyacetophenone. The compound (0.50 g, 1.79 mmol) and zinc chloride (1.21 g, 0.89 mol) were heated in ethanol (10 ml) for an hour along with a small quantity (0.02 g) of sodium hydride. The compound was recrystallized from ethanol.

### Refinement

The ethylene linkage in the free Schiff base is disordered over two position; these were arbitrarily assigned 0.5 site occupancies; the temperature factors of the primed atoms were set to those of the unprimed atoms. The N3–C27 and N3–C27' were restrained to within 0.01 Å of each other; the four C–C bonds were restrained to 1.50±0.01 Å.

Carbon-bound H-atoms were placed in calculated positions (C—H 0.93 to 0.98 Å) and were included in the refinement in the riding model approximation, with *U*(H) set to 1.2 to 1.5*U*(C). The amino H-atoms were located in a difference Fourier map, and were refined with an N–H distance restraint of 0.86±0.01 Å; their temperature factors were freely refined.

### Crystal data

**Table:**

[ZnCl_2_(C_18_H_18_N_2_O)_2_]·2C_18_H_18_N_2_O	*F*_000_ = 1312
*M**_r_* = 1249.65	*D*_x_ = 1.325 Mg m^−^^3^
Monoclinic, *C*2	Mo *K*α radiation λ = 0.71073 Å
Hall symbol: C 2y	Cell parameters from 8552 reflections
*a* = 25.8073 (4) Å	θ = 2.4–23.8º
*b* = 9.1754 (1) Å	µ = 0.54 mm^−^^1^
*c* = 14.3265 (2) Å	*T* = 295 (2) K
β = 112.566 (1)º	Irregular block, yellow
*V* = 3132.67 (7) Å^3^	0.10 × 0.06 × 0.05 mm
*Z* = 2	

### Data collection

**Table:**

Bruker SMART APEXII diffractometer	7190 independent reflections
Radiation source: fine-focus sealed tube	6008 reflections with *I* > 2σ(*I*)
Monochromator: graphite	*R*_int_ = 0.039
*T* = 295(2) K	θ_max_ = 27.5º
φ and ω scans	θ_min_ = 1.5º
Absorption correction: multi-scan(SADABS; Sheldrick, 1996)	*h* = −33→32
*T*_min_ = 0.866, *T*_max_ = 0.974	*k* = −11→11
36333 measured reflections	*l* = −18→18

### Refinement

**Table:**

Refinement on *F*^2^	Hydrogen site location: inferred from neighbouring sites
Least-squares matrix: full	H atoms treated by a mixture of independent and constrained refinement
*R*[*F*^2^ > 2σ(*F*^2^)] = 0.037	*w* = 1/[σ^2^(*F*_o_^2^) + (0.0481*P*)^2^ + 0.6205*P*] where *P* = (*F*_o_^2^ + 2*F*_c_^2^)/3
*wR*(*F*^2^) = 0.091	(Δ/σ)_max_ = 0.001
*S* = 1.00	Δρ_max_ = 0.36 e Å^−^^3^
7190 reflections	Δρ_min_ = −0.30 e Å^−^^3^
413 parameters	Extinction correction: none
10 restraints	Absolute structure: Flack (1983), 3669 Friedel pairs
Primary atom site location: structure-invariant direct methods	Flack parameter: 0.000 (8)
Secondary atom site location: difference Fourier map	

### Fractional atomic coordinates and isotropic or equivalent isotropic displacement parameters (Å^2^)

**Table:**

	*x*	*y*	*z*	*U*_iso_*/*U*_eq_	Occ. (<1)
Zn1	0.5000	0.50003 (3)	0.0000	0.03995 (10)	
Cl1	0.42528 (3)	0.63088 (6)	−0.09253 (5)	0.05459 (17)	
O1	0.48035 (7)	0.36059 (16)	0.08739 (12)	0.0449 (4)	
O2	0.42302 (10)	0.7684 (3)	0.31515 (19)	0.0846 (7)	
N1	0.39707 (9)	0.1849 (2)	0.02989 (16)	0.0455 (5)	
H1N	0.4136 (10)	0.2679 (17)	0.034 (2)	0.055*	
N2	0.34836 (9)	0.5756 (2)	0.16913 (19)	0.0602 (6)	
H2N	0.3665 (11)	0.646 (2)	0.2068 (19)	0.072*	
N3	0.52785 (12)	0.7793 (3)	0.3958 (2)	0.0850 (9)	
H3N	0.4975 (11)	0.731 (4)	0.388 (3)	0.102*	
N4	0.67920 (12)	0.2805 (3)	0.5240 (2)	0.0729 (7)	
H4N	0.6967 (13)	0.221 (3)	0.5714 (19)	0.087*	
C1	0.51286 (10)	0.2466 (2)	0.12619 (16)	0.0387 (5)	
C2	0.57066 (11)	0.2636 (3)	0.18025 (18)	0.0492 (6)	
H2	0.5858	0.3570	0.1916	0.059*	
C3	0.60560 (11)	0.1461 (3)	0.2170 (2)	0.0541 (6)	
H3	0.6438	0.1611	0.2525	0.065*	
C4	0.58430 (10)	0.0056 (4)	0.20174 (17)	0.0552 (6)	
H4	0.6081	−0.0740	0.2250	0.066*	
C5	0.52764 (11)	−0.0147 (3)	0.15191 (17)	0.0499 (6)	
H5	0.5134	−0.1090	0.1424	0.060*	
C6	0.49053 (10)	0.1031 (2)	0.11484 (16)	0.0386 (5)	
C7	0.43083 (10)	0.0765 (3)	0.06467 (18)	0.0424 (6)	
C8	0.40747 (13)	−0.0742 (3)	0.0536 (2)	0.0578 (7)	
H8A	0.3680	−0.0695	0.0396	0.087*	
H8B	0.4262	−0.1274	0.1151	0.087*	
H8C	0.4132	−0.1226	−0.0011	0.087*	
C9	0.33622 (11)	0.1796 (3)	−0.0260 (2)	0.0558 (7)	
H9A	0.3186	0.1324	0.0148	0.067*	
H9B	0.3277	0.1231	−0.0874	0.067*	
C10	0.31327 (11)	0.3332 (3)	−0.0526 (2)	0.0588 (7)	
H10A	0.3338	0.3826	−0.0877	0.071*	
H10B	0.2742	0.3278	−0.0984	0.071*	
C11	0.31755 (10)	0.4211 (3)	0.03838 (19)	0.0480 (6)	
C12	0.35561 (10)	0.5266 (3)	0.0851 (2)	0.0553 (7)	
H12	0.3828	0.5606	0.0629	0.066*	
C13	0.28431 (9)	0.4020 (2)	0.09671 (18)	0.0446 (5)	
C14	0.23988 (10)	0.3105 (3)	0.0911 (2)	0.0571 (7)	
H14	0.2253	0.2449	0.0379	0.069*	
C15	0.21779 (12)	0.3177 (4)	0.1644 (3)	0.0705 (9)	
H15	0.1883	0.2565	0.1604	0.085*	
C16	0.23887 (14)	0.4148 (4)	0.2439 (3)	0.0765 (10)	
H16	0.2231	0.4177	0.2924	0.092*	
C17	0.28259 (11)	0.5073 (4)	0.2531 (2)	0.0671 (7)	
H17	0.2967	0.5719	0.3070	0.081*	
C18	0.30495 (9)	0.5006 (3)	0.17897 (18)	0.0499 (5)	
C19	0.42330 (12)	0.9098 (3)	0.31308 (19)	0.0585 (7)	
C20	0.37283 (12)	0.9902 (5)	0.2698 (2)	0.0741 (9)	
H20	0.3388	0.9407	0.2442	0.089*	
C21	0.37302 (18)	1.1387 (5)	0.2649 (2)	0.0867 (12)	
H21	0.3391	1.1882	0.2358	0.104*	
C22	0.4216 (2)	1.2159 (5)	0.3016 (3)	0.0863 (12)	
H22	0.4210	1.3170	0.2974	0.104*	
C23	0.47149 (15)	1.1436 (3)	0.3450 (2)	0.0668 (8)	
H23	0.5047	1.1968	0.3701	0.080*	
C24	0.47389 (10)	0.9900 (4)	0.35243 (16)	0.0504 (6)	
C25	0.52729 (12)	0.9180 (4)	0.3974 (2)	0.0599 (7)	
C26	0.58061 (12)	1.0014 (6)	0.4431 (2)	0.0827 (9)	
H26A	0.6118	0.9377	0.4533	0.124*	
H26B	0.5834	1.0412	0.5069	0.124*	
H26C	0.5810	1.0793	0.3986	0.124*	
C27	0.5727 (6)	0.672 (2)	0.4535 (10)	0.089 (4)	0.50
H27A	0.6085	0.7217	0.4843	0.107*	0.50
H27B	0.5636	0.6270	0.5065	0.107*	0.50
C28	0.5756 (7)	0.560 (2)	0.3803 (13)	0.070 (3)	0.50
H28A	0.5819	0.6077	0.3251	0.084*	0.50
H28B	0.5401	0.5087	0.3524	0.084*	0.50
C27'	0.5826 (6)	0.7006 (19)	0.4208 (10)	0.089 (4)	0.50
H27C	0.6029	0.7411	0.3822	0.107*	0.50
H27D	0.6057	0.7110	0.4921	0.107*	0.50
C28'	0.5698 (6)	0.544 (2)	0.3950 (14)	0.070 (3)	0.50
H28C	0.5510	0.5343	0.3223	0.084*	0.50
H28D	0.5446	0.5093	0.4257	0.084*	0.50
C29	0.62217 (12)	0.4534 (3)	0.4312 (2)	0.0615 (7)	
C30	0.63045 (14)	0.3544 (4)	0.5058 (2)	0.0734 (9)	
H30	0.6065	0.3392	0.5394	0.088*	
C31	0.66965 (11)	0.4438 (3)	0.40353 (18)	0.0511 (6)	
C32	0.68572 (11)	0.5145 (3)	0.33287 (18)	0.0600 (7)	
H32	0.6629	0.5859	0.2911	0.072*	
C33	0.73566 (13)	0.4775 (4)	0.3255 (2)	0.0691 (8)	
H33	0.7470	0.5266	0.2798	0.083*	
C34	0.76939 (14)	0.3683 (4)	0.3852 (3)	0.0743 (9)	
H34	0.8028	0.3447	0.3782	0.089*	
C35	0.75462 (13)	0.2946 (3)	0.4541 (2)	0.0708 (9)	
H35	0.7772	0.2211	0.4938	0.085*	
C36	0.70474 (11)	0.3333 (3)	0.4627 (2)	0.0547 (7)	

### Atomic displacement parameters (Å^2^)

**Table:**

	*U*^11^	*U*^22^	*U*^33^	*U*^12^	*U*^13^	*U*^23^
Zn1	0.04012 (19)	0.02675 (16)	0.0496 (2)	0.000	0.01350 (16)	0.000
Cl1	0.0518 (4)	0.0378 (3)	0.0632 (4)	0.0119 (3)	0.0098 (3)	0.0003 (3)
O1	0.0435 (9)	0.0329 (7)	0.0567 (10)	−0.0008 (7)	0.0175 (8)	0.0039 (7)
O2	0.0729 (15)	0.0688 (15)	0.0839 (16)	−0.0066 (12)	−0.0012 (13)	−0.0150 (12)
N1	0.0477 (12)	0.0389 (10)	0.0508 (12)	−0.0063 (9)	0.0199 (10)	−0.0071 (9)
N2	0.0487 (12)	0.0463 (12)	0.0773 (16)	−0.0069 (10)	0.0150 (12)	−0.0127 (11)
N3	0.0659 (17)	0.0699 (18)	0.0893 (19)	0.0228 (14)	−0.0037 (15)	−0.0205 (15)
N4	0.0775 (18)	0.0613 (15)	0.0666 (17)	0.0077 (14)	0.0130 (14)	0.0143 (13)
C1	0.0482 (13)	0.0333 (11)	0.0359 (11)	−0.0014 (9)	0.0176 (10)	0.0008 (9)
C2	0.0507 (14)	0.0436 (12)	0.0486 (13)	−0.0034 (11)	0.0140 (11)	0.0014 (11)
C3	0.0478 (14)	0.0616 (16)	0.0481 (14)	0.0046 (12)	0.0132 (12)	0.0063 (12)
C4	0.0586 (14)	0.0498 (13)	0.0556 (13)	0.0180 (16)	0.0203 (11)	0.0127 (15)
C5	0.0703 (16)	0.0344 (12)	0.0517 (13)	0.0056 (13)	0.0310 (12)	0.0038 (11)
C6	0.0501 (13)	0.0326 (10)	0.0379 (11)	−0.0004 (10)	0.0223 (10)	−0.0021 (9)
C7	0.0588 (15)	0.0348 (12)	0.0398 (12)	−0.0054 (11)	0.0257 (11)	−0.0087 (10)
C8	0.0727 (19)	0.0385 (13)	0.0617 (16)	−0.0087 (13)	0.0255 (15)	−0.0106 (12)
C9	0.0436 (14)	0.0582 (16)	0.0607 (16)	−0.0075 (12)	0.0145 (12)	−0.0154 (13)
C10	0.0482 (15)	0.0677 (17)	0.0509 (15)	0.0049 (13)	0.0084 (12)	−0.0019 (13)
C11	0.0375 (12)	0.0447 (13)	0.0550 (14)	0.0059 (10)	0.0101 (11)	0.0052 (11)
C12	0.0438 (13)	0.0489 (17)	0.0743 (17)	−0.0022 (11)	0.0238 (12)	0.0045 (13)
C13	0.0348 (12)	0.0366 (11)	0.0531 (14)	0.0066 (9)	0.0066 (10)	0.0057 (10)
C14	0.0355 (13)	0.0498 (14)	0.0729 (18)	0.0018 (11)	0.0060 (13)	0.0089 (13)
C15	0.0380 (14)	0.075 (2)	0.095 (2)	0.0076 (14)	0.0222 (15)	0.0353 (19)
C16	0.0598 (19)	0.102 (3)	0.076 (2)	0.0266 (19)	0.0349 (17)	0.031 (2)
C17	0.0615 (16)	0.0736 (18)	0.0622 (15)	0.0206 (19)	0.0192 (13)	0.0061 (18)
C18	0.0412 (11)	0.0427 (10)	0.0610 (13)	0.0091 (13)	0.0144 (10)	0.0040 (14)
C19	0.0562 (16)	0.0687 (18)	0.0448 (14)	0.0091 (14)	0.0130 (13)	−0.0077 (13)
C20	0.0525 (15)	0.112 (3)	0.0531 (15)	0.023 (2)	0.0152 (12)	0.005 (2)
C21	0.091 (3)	0.113 (3)	0.0546 (19)	0.056 (3)	0.0264 (19)	0.016 (2)
C22	0.125 (4)	0.067 (2)	0.071 (2)	0.039 (2)	0.043 (3)	0.0180 (18)
C23	0.085 (2)	0.0625 (18)	0.0589 (17)	0.0018 (16)	0.0335 (16)	−0.0014 (14)
C24	0.0519 (13)	0.0588 (14)	0.0414 (11)	0.0137 (15)	0.0191 (10)	−0.0012 (13)
C25	0.0546 (16)	0.0717 (18)	0.0478 (14)	0.0089 (14)	0.0134 (12)	−0.0180 (13)
C26	0.0597 (17)	0.117 (3)	0.0654 (17)	−0.004 (2)	0.0172 (14)	−0.019 (2)
C27	0.092 (5)	0.085 (7)	0.060 (8)	0.042 (6)	−0.005 (4)	−0.012 (5)
C28	0.062 (3)	0.076 (4)	0.067 (5)	0.018 (3)	0.019 (2)	−0.008 (3)
C27'	0.092 (5)	0.085 (7)	0.060 (8)	0.042 (6)	−0.005 (4)	−0.012 (5)
C28'	0.062 (3)	0.076 (4)	0.067 (5)	0.018 (3)	0.019 (2)	−0.008 (3)
C29	0.0613 (17)	0.0576 (16)	0.0543 (15)	0.0111 (13)	0.0097 (13)	−0.0100 (12)
C30	0.070 (2)	0.075 (2)	0.072 (2)	−0.0018 (17)	0.0247 (17)	−0.0056 (17)
C31	0.0500 (14)	0.0413 (11)	0.0441 (13)	0.0061 (11)	−0.0018 (11)	−0.0081 (10)
C32	0.0681 (16)	0.0497 (14)	0.0455 (12)	0.0093 (15)	0.0032 (11)	−0.0018 (13)
C33	0.0711 (18)	0.070 (2)	0.0570 (15)	−0.0006 (16)	0.0145 (14)	−0.0038 (15)
C34	0.0569 (18)	0.079 (2)	0.076 (2)	0.0046 (16)	0.0129 (16)	−0.0055 (18)
C35	0.0528 (17)	0.0591 (17)	0.079 (2)	0.0162 (14)	0.0013 (15)	0.0040 (15)
C36	0.0544 (16)	0.0406 (14)	0.0524 (15)	0.0036 (12)	0.0019 (12)	0.0017 (11)

### Geometric parameters (Å, °)

**Table:**

Zn1—O1^i^	1.987 (2)	C15—C16	1.383 (5)
Zn1—O1	1.987 (2)	C15—H15	0.9300
Zn1—Cl1^i^	2.2260 (6)	C16—C17	1.377 (5)
Zn1—Cl1	2.2260 (6)	C16—H16	0.9300
O1—C1	1.323 (3)	C17—C18	1.391 (3)
O2—C19	1.298 (4)	C17—H17	0.9300
N1—C7	1.290 (3)	C19—C24	1.414 (4)
N1—C9	1.464 (3)	C19—C20	1.416 (4)
N1—H1N	0.86 (1)	C20—C21	1.365 (6)
N2—C12	1.363 (4)	C20—H20	0.9300
N2—C18	1.367 (3)	C21—C22	1.358 (6)
N2—H2N	0.86 (1)	C21—H21	0.9300
N3—C25	1.273 (4)	C22—C23	1.367 (5)
N3—C27	1.500 (7)	C22—H22	0.9300
N3—C27'	1.502 (7)	C23—C24	1.413 (4)
N3—H3N	0.87 (1)	C23—H23	0.9300
N4—C30	1.364 (4)	C24—C25	1.438 (4)
N4—C36	1.373 (4)	C25—C26	1.489 (5)
N4—H4N	0.86 (1)	C26—H26A	0.9600
C1—C2	1.401 (3)	C26—H26B	0.9600
C1—C6	1.421 (3)	C26—H26C	0.9600
C2—C3	1.374 (4)	C27—C28	1.491 (9)
C2—H2	0.9300	C27—H27A	0.9700
C3—C4	1.386 (4)	C27—H27B	0.9700
C3—H3	0.9300	C28—C29	1.506 (8)
C4—C5	1.372 (3)	C28—H28A	0.9700
C4—H4	0.9300	C28—H28B	0.9700
C5—C6	1.406 (3)	C27'—C28'	1.485 (9)
C5—H5	0.9300	C27'—H27C	0.9700
C6—C7	1.449 (3)	C27'—H27D	0.9700
C7—C8	1.492 (3)	C28'—C29	1.502 (8)
C8—H8A	0.9600	C28'—H28C	0.9700
C8—H8B	0.9600	C28'—H28D	0.9700
C8—H8C	0.9600	C29—C30	1.355 (4)
C9—C10	1.520 (4)	C29—C31	1.428 (4)
C9—H9A	0.9700	C30—H30	0.9300
C9—H9B	0.9700	C31—C32	1.392 (4)
C10—C11	1.500 (4)	C31—C36	1.406 (3)
C10—H10A	0.9700	C32—C33	1.375 (4)
C10—H10B	0.9700	C32—H32	0.9300
C11—C12	1.358 (3)	C33—C34	1.386 (4)
C11—C13	1.420 (4)	C33—H33	0.9300
C12—H12	0.9300	C34—C35	1.365 (5)
C13—C14	1.398 (3)	C34—H34	0.9300
C13—C18	1.418 (4)	C35—C36	1.386 (4)
C14—C15	1.374 (4)	C35—H35	0.9300
C14—H14	0.9300		
			
O1—Zn1—O1^i^	99.84 (9)	N2—C18—C17	130.8 (3)
O1—Zn1—Cl1	110.51 (5)	N2—C18—C13	107.3 (2)
O1^i^—Zn1—Cl1^i^	110.51 (5)	C17—C18—C13	122.0 (3)
O1—Zn1—Cl1^i^	110.14 (5)	O2—C19—C24	121.6 (3)
O1^i^—Zn1—Cl1	110.14 (5)	O2—C19—C20	121.2 (3)
Cl1—Zn1—Cl1^i^	114.72 (3)	C24—C19—C20	117.2 (3)
C1—O1—Zn1	120.01 (14)	C21—C20—C19	121.5 (4)
C7—N1—C9	127.5 (2)	C21—C20—H20	119.3
C7—N1—H1N	114 (2)	C19—C20—H20	119.3
C9—N1—H1N	118 (2)	C20—C21—C22	121.4 (3)
C12—N2—C18	108.7 (2)	C20—C21—H21	119.3
C12—N2—H2N	125 (2)	C22—C21—H21	119.3
C18—N2—H2N	126 (2)	C21—C22—C23	119.5 (4)
C25—N3—C27	130.9 (9)	C21—C22—H22	120.3
C25—N3—C27'	119.5 (9)	C23—C22—H22	120.3
C27—N3—C27'	25.6 (9)	C22—C23—C24	121.6 (3)
C25—N3—H3N	120 (3)	C22—C23—H23	119.2
C27—N3—H3N	102 (3)	C24—C23—H23	119.2
C27'—N3—H3N	121 (3)	C19—C24—C23	118.8 (3)
C30—N4—C36	109.6 (3)	C19—C24—C25	121.3 (3)
C30—N4—H4N	130 (2)	C23—C24—C25	119.9 (3)
C36—N4—H4N	120 (2)	N3—C25—C24	117.8 (3)
O1—C1—C2	120.9 (2)	N3—C25—C26	120.5 (3)
O1—C1—C6	121.4 (2)	C24—C25—C26	121.7 (3)
C2—C1—C6	117.8 (2)	C25—C26—H26A	109.5
C3—C2—C1	121.8 (2)	C25—C26—H26B	109.5
C3—C2—H2	119.1	H26A—C26—H26B	109.5
C1—C2—H2	119.1	C25—C26—H26C	109.5
C2—C3—C4	120.5 (2)	H26A—C26—H26C	109.5
C2—C3—H3	119.8	H26B—C26—H26C	109.5
C4—C3—H3	119.8	C28—C27—N3	107.4 (8)
C5—C4—C3	119.2 (3)	C28—C27—H27A	110.2
C5—C4—H4	120.4	N3—C27—H27A	110.2
C3—C4—H4	120.4	C28—C27—H27B	110.2
C4—C5—C6	121.9 (3)	N3—C27—H27B	110.2
C4—C5—H5	119.1	H27A—C27—H27B	108.5
C6—C5—H5	119.1	C27—C28—C29	110.8 (10)
C5—C6—C1	118.8 (2)	C27—C28—H28A	109.5
C5—C6—C7	119.9 (2)	C29—C28—H28A	109.5
C1—C6—C7	121.3 (2)	C27—C28—H28B	109.5
N1—C7—C6	119.6 (2)	C29—C28—H28B	109.5
N1—C7—C8	119.2 (2)	H28A—C28—H28B	108.1
C6—C7—C8	121.2 (2)	C28'—C27'—N3	107.8 (10)
C7—C8—H8A	109.5	C28'—C27'—H27C	110.2
C7—C8—H8B	109.5	N3—C27'—H27C	110.2
H8A—C8—H8B	109.5	C28'—C27'—H27D	110.2
C7—C8—H8C	109.5	N3—C27'—H27D	110.2
H8A—C8—H8C	109.5	H27C—C27'—H27D	108.5
H8B—C8—H8C	109.5	C27'—C28'—C29	111.4 (11)
N1—C9—C10	109.8 (2)	C27'—C28'—H28C	109.3
N1—C9—H9A	109.7	C29—C28'—H28C	109.3
C10—C9—H9A	109.7	C27'—C28'—H28D	109.3
N1—C9—H9B	109.7	C29—C28'—H28D	109.3
C10—C9—H9B	109.7	H28C—C28'—H28D	108.0
H9A—C9—H9B	108.2	C30—C29—C31	106.5 (3)
C11—C10—C9	112.9 (2)	C30—C29—C28	132.3 (8)
C11—C10—H10A	109.0	C31—C29—C28	121.1 (8)
C9—C10—H10A	109.0	C30—C29—C28'	119.6 (8)
C11—C10—H10B	109.0	C31—C29—C28'	133.9 (8)
C9—C10—H10B	109.0	C28—C29—C28'	12.8 (15)
H10A—C10—H10B	107.8	C29—C30—N4	109.8 (3)
C12—C11—C13	106.5 (2)	C29—C30—H30	125.1
C12—C11—C10	127.6 (3)	N4—C30—H30	125.1
C13—C11—C10	125.8 (2)	C32—C31—C36	118.1 (3)
C11—C12—N2	110.6 (2)	C32—C31—C29	134.4 (2)
C11—C12—H12	124.7	C36—C31—C29	107.5 (3)
N2—C12—H12	124.7	C33—C32—C31	119.4 (3)
C14—C13—C18	118.0 (2)	C33—C32—H32	120.3
C14—C13—C11	135.1 (2)	C31—C32—H32	120.3
C18—C13—C11	106.9 (2)	C32—C33—C34	121.1 (3)
C15—C14—C13	119.9 (3)	C32—C33—H33	119.5
C15—C14—H14	120.0	C34—C33—H33	119.5
C13—C14—H14	120.0	C35—C34—C33	121.3 (3)
C14—C15—C16	120.8 (3)	C35—C34—H34	119.3
C14—C15—H15	119.6	C33—C34—H34	119.3
C16—C15—H15	119.6	C34—C35—C36	117.6 (3)
C17—C16—C15	121.6 (3)	C34—C35—H35	121.2
C17—C16—H16	119.2	C36—C35—H35	121.2
C15—C16—H16	119.2	N4—C36—C35	131.0 (3)
C16—C17—C18	117.7 (3)	N4—C36—C31	106.5 (2)
C16—C17—H17	121.2	C35—C36—C31	122.5 (3)
C18—C17—H17	121.2		
			
O1^i^—Zn1—O1—C1	44.94 (13)	C21—C22—C23—C24	0.1 (5)
Cl1^i^—Zn1—O1—C1	−71.31 (16)	O2—C19—C24—C23	178.0 (3)
Cl1—Zn1—O1—C1	160.91 (14)	C20—C19—C24—C23	−1.1 (4)
Zn1—O1—C1—C2	52.2 (3)	O2—C19—C24—C25	−0.6 (4)
Zn1—O1—C1—C6	−127.91 (18)	C20—C19—C24—C25	−179.7 (2)
O1—C1—C2—C3	−176.8 (2)	C22—C23—C24—C19	0.6 (4)
C6—C1—C2—C3	3.3 (4)	C22—C23—C24—C25	179.2 (3)
C1—C2—C3—C4	−0.2 (4)	C27—N3—C25—C24	−165.0 (6)
C2—C3—C4—C5	−1.9 (4)	C27'—N3—C25—C24	166.8 (6)
C3—C4—C5—C6	0.7 (4)	C27—N3—C25—C26	15.9 (8)
C4—C5—C6—C1	2.4 (3)	C27'—N3—C25—C26	−12.3 (7)
C4—C5—C6—C7	−178.6 (2)	C19—C24—C25—N3	3.7 (4)
O1—C1—C6—C5	175.8 (2)	C23—C24—C25—N3	−174.9 (3)
C2—C1—C6—C5	−4.3 (3)	C19—C24—C25—C26	−177.3 (2)
O1—C1—C6—C7	−3.1 (3)	C23—C24—C25—C26	4.2 (4)
C2—C1—C6—C7	176.7 (2)	C25—N3—C27—C28	−135.0 (9)
C9—N1—C7—C6	177.4 (2)	C27'—N3—C27—C28	−63 (3)
C9—N1—C7—C8	−3.4 (4)	N3—C27—C28—C29	176.6 (14)
C5—C6—C7—N1	−179.3 (2)	C25—N3—C27'—C28'	−169.8 (9)
C1—C6—C7—N1	−0.3 (3)	C27—N3—C27'—C28'	66 (4)
C5—C6—C7—C8	1.5 (4)	N3—C27'—C28'—C29	−171.8 (10)
C1—C6—C7—C8	−179.5 (2)	C27—C28—C29—C30	64.7 (16)
C7—N1—C9—C10	−179.8 (2)	C27—C28—C29—C31	−114.6 (11)
N1—C9—C10—C11	−67.5 (3)	C27—C28—C29—C28'	69 (7)
C9—C10—C11—C12	102.8 (3)	C27'—C28'—C29—C30	113.1 (11)
C9—C10—C11—C13	−72.5 (3)	C27'—C28'—C29—C31	−67.4 (16)
C13—C11—C12—N2	−0.2 (3)	C27'—C28'—C29—C28	−64 (7)
C10—C11—C12—N2	−176.2 (2)	C31—C29—C30—N4	−2.0 (3)
C18—N2—C12—C11	0.5 (3)	C28—C29—C30—N4	178.6 (12)
C12—C11—C13—C14	−178.4 (3)	C28'—C29—C30—N4	177.6 (10)
C10—C11—C13—C14	−2.3 (4)	C36—N4—C30—C29	1.6 (4)
C12—C11—C13—C18	−0.1 (3)	C30—C29—C31—C32	179.5 (3)
C10—C11—C13—C18	176.0 (2)	C28—C29—C31—C32	−1.0 (11)
C18—C13—C14—C15	−0.3 (3)	C28'—C29—C31—C32	0.0 (13)
C11—C13—C14—C15	177.8 (3)	C30—C29—C31—C36	1.6 (3)
C13—C14—C15—C16	0.1 (4)	C28—C29—C31—C36	−178.9 (10)
C14—C15—C16—C17	−0.2 (4)	C28'—C29—C31—C36	−177.9 (12)
C15—C16—C17—C18	0.3 (4)	C36—C31—C32—C33	−2.0 (4)
C12—N2—C18—C17	177.8 (3)	C29—C31—C32—C33	−179.8 (3)
C12—N2—C18—C13	−0.5 (3)	C31—C32—C33—C34	1.9 (4)
C16—C17—C18—N2	−178.6 (3)	C32—C33—C34—C35	−0.7 (5)
C16—C17—C18—C13	−0.5 (4)	C33—C34—C35—C36	−0.3 (5)
C14—C13—C18—N2	179.0 (2)	C30—N4—C36—C35	179.4 (3)
C11—C13—C18—N2	0.4 (3)	C30—N4—C36—C31	−0.6 (3)
C14—C13—C18—C17	0.5 (4)	C34—C35—C36—N4	−179.9 (3)
C11—C13—C18—C17	−178.1 (2)	C34—C35—C36—C31	0.1 (4)
O2—C19—C20—C21	−178.1 (3)	C32—C31—C36—N4	−179.0 (2)
C24—C19—C20—C21	0.9 (4)	C29—C31—C36—N4	−0.6 (3)
C19—C20—C21—C22	−0.2 (5)	C32—C31—C36—C35	1.1 (4)
C20—C21—C22—C23	−0.3 (5)	C29—C31—C36—C35	179.4 (3)

Symmetry codes: (i) −*x*+1, *y*, −*z*.

### Hydrogen-bond geometry (Å, °)

**Table:**

*D*—H···*A*	*D*—H	H···*A*	*D*···*A*	*D*—H···*A*
N1—H1*n*···O1	0.86 (1)	1.81 (2)	2.557 (3)	144 (3)
N2—H2*n*···O2	0.86 (1)	2.01 (1)	2.851 (3)	164 (3)
N3—H3*n*···O2	0.87 (1)	1.83 (3)	2.503 (4)	133 (4)
